# Compatibility of the food compass with dietary indices and sustainable eating behaviors: toward a multidimensional assessment tool

**DOI:** 10.1186/s41043-026-01410-x

**Published:** 2026-07-28

**Authors:** Nazlıcan Erdoğan Gövez, Emine Özdemir, Vesile Dinç, Hayrunnisa Ayhan, Eda Köksal

**Affiliations:** Department of Nutrition and Dietetics, Faculty of Health Sciences, Gazi University, Ankara, Türkiye

**Keywords:** Food compass, Diet quality, Dietary index, Healthy eating index, Sustainable nutrition, Mediterranean diet

## Abstract

**Background:**

Non-communicable diseases remain the leading cause of global mortality, with diet being a major modifiable risk factor. The Food Compass (FC), a novel nutrient profiling system, offers a broader perspective. This study aimed to assess the correlation between a Dietary Index (DI) derived from Food Compass and two established indices Healthy Eating Index (HEI) and the Mediterranean Adequacy Index (MAI), and to examine its association with sustainable and healthy eating behaviors (SHEB).

**Methods:**

In this cross-sectional study, 294 participants completed a questionnaire to collect sociodemographic information, underwent anthropometric measurements, and provided 24-hour dietary recalls. From these dietary recalls, HEI, MAI, and DI scores were calculated. SHEB was assessed using a validated scale.

**Results:**

The DI showed significant correlations with HEI (*r* = 0.20, *p* < 0.01) and MAI (*r* = 0.46, *p* < 0.01). Only the DI demonstrated a significant association with SHEB (*r* = 0.17, *p* < 0.01). Bland–Altman analyses revealed overall agreement between the indices, with HEI showing relatively homogeneous differences from the DI. Regression analysis confirmed DI as an independent predictor of SHEBS (β = 0.12, *p* = 0.034).

**Conclusions:**

FC may serve as a novel, multidimensional dietary assessment tool that better captures sustainable eating behaviors compared to traditional indices.

**Supplementary Information:**

The online version contains supplementary material available at https://doi.org/10.1186/s41043-026-01410-x.

## Introduction

Non-communicable diseases (NCDs) constitute the primary cause of worldwide mortality, responsible for over 74% of all fatalities, with diet identified as a significant modifiable risk factor [1]. High diet quality is associated with an optimal nutrient intake profile and a substantially reduced risk of diet-related NCDs [2].

Evaluating the relationship between health outcomes and overall dietary patterns, rather than focusing on specific nutrients, is a crucial element of nutritional epidemiology and public health [3]. The overall diet can be assessed by diet quality indices (DQIs), where a higher score generally indicates superior diet quality or compliance with dietary guidelines [4]. Early dietary quality indices, such as the Healthy Eating Index (HEI) [5], were developed based on the American Dietary Guidelines, whereas the Mediterranean Adequacy Index (MAI) [6] was established following the principles of the traditional Mediterranean dietary pattern.

More recently, nutrient profiling systems (NPSs) have garnered growing attention in public health research as tools for guiding healthier food choices. Defined as the scientific method of classifying or ranking foods according to their nutrient composition to promote health and preventing disease, NPSs typically use quantitative algorithms that evaluate foods based on the presence and/or amount of both beneficial components (such as specific vitamins or fruit content) and adverse components (energy, saturated fat, salt, added sugars etc.) [7]. The Food Compass (FC) [8] was developed to incorporate a broader spectrum of food characteristics and to provide a standardized scoring system. It evaluates nine health-relevant domains, including nutrient ratios, vitamins, minerals, food ingredients, additives, processing level, fat, fiber and protein, and phytochemicals. By accounting for these multidimensional factors, FC offers a broader perspective, capturing the complexity of modern diets while aligning with sustainable eating principles through its emphasis on plant-based and minimally processed foods.

To date, limited research has evaluated how FC aligns with established DQIs [9–11] or its relationship with environmental aspects [12, 13]. This study aims to assess the correlation between the Dietary Index (DI) derived from Food Compass and two established dietary indices, HEI and MAI, and to examine its association with sustainable and healthy eating behaviors. Given that FC is a novel NPS, we aimed to evaluate its usability and its capacity to encompass dimensions of diet quality that extend beyond those represented by conventional indices.

## Material and methods

### Study population and design

This cross-sectional study was conducted between March 2024 and April 2025 and included a total of 294 (100 males and 194 females) adults with a mean age of 29.3 ± 11.82 years who voluntarily participated through convenience sampling due to ease of access for participants. Individuals with implausible energy intake levels (i.e., < 500 kcal/day or > 3500 kcal/day for women; <800 kcal/day or > 4000 kcal/day for men), those following a specific diet, individuals with severe cognitive impairment, or those with communication difficulties were excluded from the study.

A questionnaire developed by the researchers was administered by the face-to-face method to assess participants’ sociodemographic characteristics (age, gender, marital and educational status). Height was measured to the closest 0.1 cm utilizing a portable stadiometer, with individuals standing upright, barefoot, with feet aligned, and the head positioned in the Frankfort horizontal plane. Body weight was measured using an electronic scale, and Body Mass Index (BMI) was calculated by dividing body weight (kg) by the square of height (m²). The classification was based on the World Health Organization (WHO) criteria: individuals with a BMI < 18.50 kg/m² were considered underweight; those with a BMI between 18.50 and 24.99 kg/m² were classified as normal weight; a BMI of 25.00–29.99 kg/m² indicated overweight; and a BMI ≥ 30.00 kg/m² was classified as obese [14].

### Sustainable and healthy eating behaviors scale (SHEBS)

The Sustainable and Healthy Eating Behaviors Scale was developed by Zakowska-Biemans et al. to assess sustainable and healthy eating behaviors [15]. The Turkish validity and reliability study of the scale was conducted by Köksal et al. in 2023 [16]. The scale consists of 32 items across 7 factors: *Healthy & Balanced Diet*, *Quality Labels (Regional and Organic)*, *Meat Reduction*, *Local Food and Avoiding Food Waste*, *Low Fat*, *Animal Welfare*, and *Seasonal Food*. Items are rated using a 7-point Likert scale, where participants are asked to indicate the frequency of each behavior with options ranging from “Never” [1] to “Always” [7]. Higher total scores indicate more sustainable and healthier eating behaviors [16].

### Dietary assessment

Dietary intake was assessed via 24-hour dietary recall and was collected by expert dietitians and researchers. For meals with ingredients that were not known by the participants, standard recipes from the *National Menu Planning and Implementation Guide for Mass Nutrition Systems* were used [17]. The Nutrition Information System (BeBis, version 8.2) was used to analyze data obtained from the 24-h dietary recalls, providing estimates of total daily energy, macronutrient, and micronutrient intakes.

### Healthy eating index (HEI)

Healthy Eating Index (HEI) was assessed using the Healthy Eating Index-2015 (HEI-2015), which was developed by the United States Department of Agriculture (USDA) and the National Cancer Institute. The HEI-2015 evaluates adherence to the 2015–2020 American Dietary Guidelines and includes 13 components reflecting key aspects of dietary quality (consumption of different food groups, fat, sodium, and sugar). Participants’ dietary data were analyzed and scored based on the standard HEI-2015 scoring system, with a maximum possible score of 100 indicating optimal adherence to dietary guidelines [5].

### Mediterranean adequacy index (MAI)

Adherence to the Mediterranean dietary pattern was assessed using the Mediterranean Adequacy Index (MAI), developed by Alberti-Fidanza and Fidanza (2004). In this context, the total energy derived from carbohydrate-based foods (bread, cereals, legumes, and potatoes) and protective food groups (vegetables, fresh legumes, fruits, and olive oil) was divided by the total energy obtained from animal-based foods (milk and dairy products, red meat, eggs, animal fats, and margarine) and sugary foods (beverages, cakes, added sugars, and cookies) to calculate the MAI score [6].

### Food compass and dietary index (DI)

The Food Compass Score (FCS) is a novel nutrient profiling system that evaluates foods based on 54 individual attributes, including factors such as the unsaturated to saturated fat ratio, fiber-to-carbohydrate ratio, additive content, and degree of processing. These attributes are organized into nine domains considered essential for assessing the healthfulness of foods: nutrient ratios, vitamins, minerals, food ingredients, additives, processing level, specific lipids, dietary fiber and protein, and phytochemicals [8]. Individual FCSs were derived by calculating dietary index values (a higher dietary index indicates better overall diet quality) based on energy-weighted averages, using the following formula [9, 18]:

$$\:Dietary\:index\:=\:\sum\:(FSi\:\times\:\:Ei)\:/\:\sum\:\left(Ei\right)$$

$$\:Ei\hspace{0.17em}=\hspace{0.17em}fi\:\times\:\:energy\:per\:portion$$

i: each food or beverage consumed by the participant

FSi: the corresponding food or beverage score

Ei: the average daily energy intake from that item (kcal)

fi: the daily portion frequency (portion/day)

### Statistical analyses

Statistical analyses were performed using IBM SPSS Statistics 29.0. Categorical variables were expressed as counts (n) and percentages (%), while continuous variables were summarized using means (M) and standard deviations (SD). The normality of the continuous variables was assessed using the Kolmogorov–Smirnov test.

Depending on the distribution of the data, either Pearson or Spearman correlation analysis was performed to examine the relationships between the Dietary Index (DI) and other dietary quality indices (HEI and MAI), as well as between DI and the total SHEBS score and its factors.

To assess agreement between the DI and the other indices (HEI and MAI), Bland–Altman plots were generated. Multiple linear regression analysis was performed to identify significant predictors of the SHEBS score. The level of statistical significance was set at *p* < 0.05 for all statistical tests.

## Results

A total of 294 participants were included in the study, with a mean age of 29.3 ± 11.82 years. The majority were female (66.0%), and 64.3% had secondary education. The mean body mass index (BMI) was 25.1 ± 2.64 kg/m² (Table 1.).

**Table 1:** General characteristics of the participants

Variable	*n* (%) or mean ± SD
**Age (years)** (mean ± SD)	29.3 ± 11.82
**Gender** [n (%)]	
Male	100 (34.0)
Female	194 (66.0)
**Educational level** [n (%)]	
Primary	30 (13.6)
Secondary	189 (64.3)
University or higher	65 (22.1)
**BMI** (kg/m^2^) (mean ± SD)	25.1 ± 2.64
**BMI classification** [n (%)]	
Underweight	13 (4.4)
Normal	144 (49.0)
Overweight and obese	137 (46.6)

*BMI*: Body Mass Index

The mean score for the HEI was 57.4 ± 12.55, whereas the MAI had a mean of 3.0 ± 2.96. The DI obtained from the food compass had a mean score of 62.8 ± 10.79. The mean score of the SHEBS was 3.7 ± 0.87. Among the SHEBS factors, the highest mean scores were observed for low-fat (4.5 ± 1.14), healthy and balanced diet (4.4 ± 1.09), and seasonal food and avoiding food waste factors (4.2 ± 1.0) (Table 2).

**Table 2:** Descriptive statistics for dietary index scores and SHEBS factors

Variable	Mean ± SD	[Min-Max]
**HEI**	57.4 ± 12.55	[27.2–90.1]
**MAI**	3.0 ± 2.96	[0.1–26.1]
**DI**	62.8 ± 10.79	[26.8–84.0]
**SHEBS**_**Total**_	3.7 ± 0.87	[1.23–6.4]
Quality labels (regional and organic)	3.7 ± 1.10	[1.0–6.5]
Seasonal food and avoiding food waste	4.2 ± 1.0	[1.3–6.7]
Animal welfare	3.4 ± 1.34	[1.0–7.0]
Meat reduction	3.0 ± 1.34	[1.0–7.0]
Healthy and balanced diet	4.4 ± 1.09	[1.0–7.0]
Local food	3.0 ± 1.35	[1.0–7.0]
Low fat	4.5 ± 1.14	[1.0–7.0]

*HEI*: Healthy Eating Index, *MAI*: Mediterranean Adequacy Index, *DI*: Dietary Index, *SHEBS*: Sustainable and Healthy Eating Behaviors Scale

**Fig. 1:**
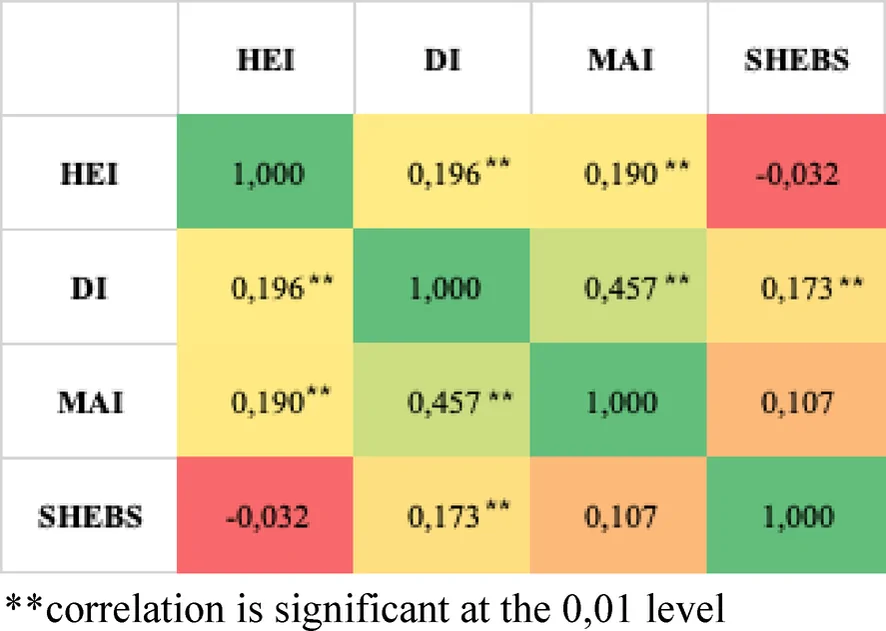
Pearson correlation matrix between HEI, MAI, DI, and SHEBS Scores. **correlation is significant at the 0,01 level

The DI showed a significant correlation with both the HEI (*r* = 0.196, *p* < 0.01) and the MAI (*r* = 0.457, *p* < 0.01). Additionally, HEI and MAI were significantly correlated with each other (*r* = 0.190, *p* < 0.01). A statistically significant association was also found between DI and the SHEBS score (*r* = 0.173, *p* < 0.01), while HEI and MAI showed no statistically significant correlation with SHEBS (*p* > 0.05) (Fig. 1).

**Fig. 2:**
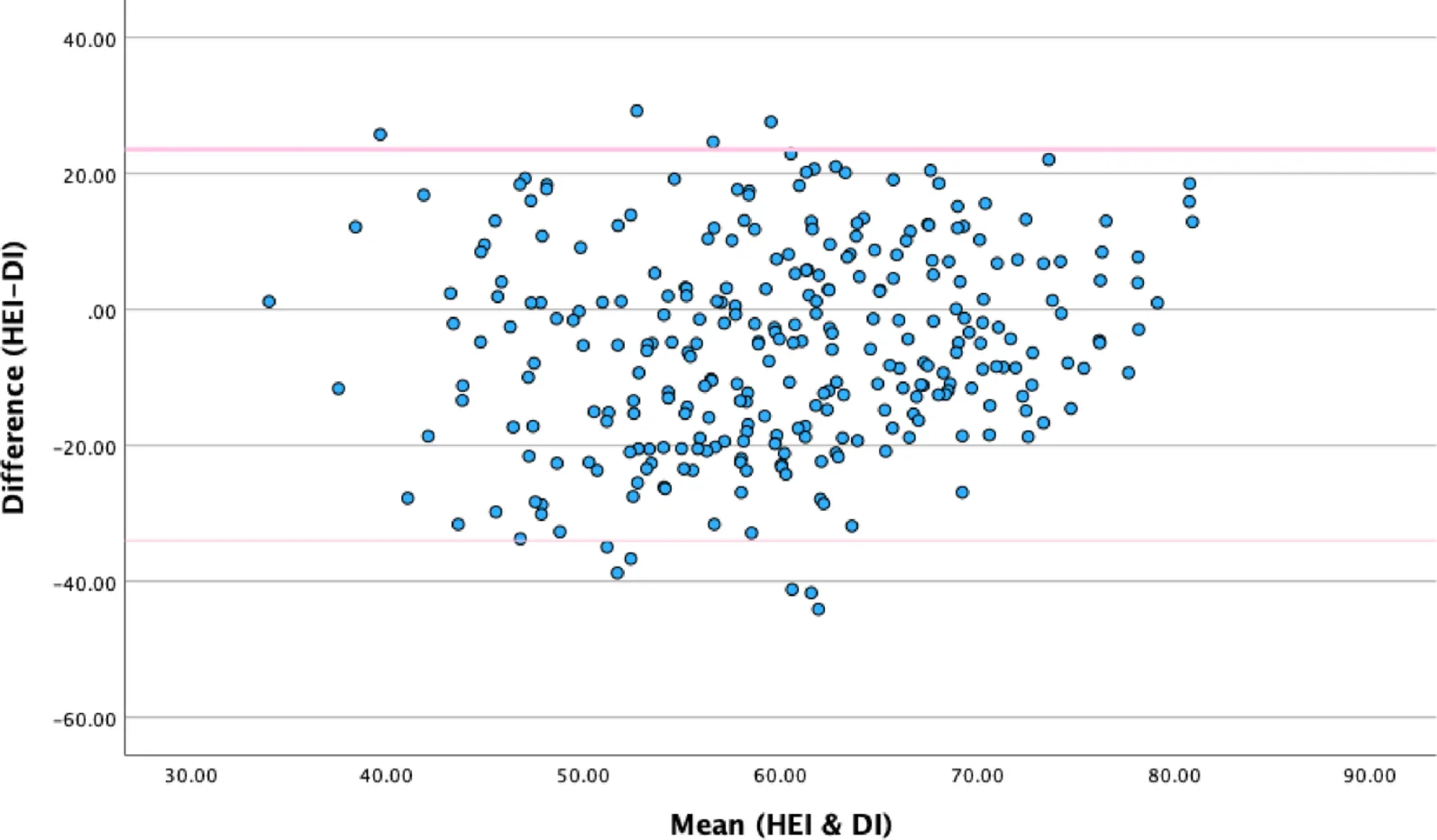
Bland-Altman plot for agreement between HEI and Food Compass (DI)

**Fig. 3:**
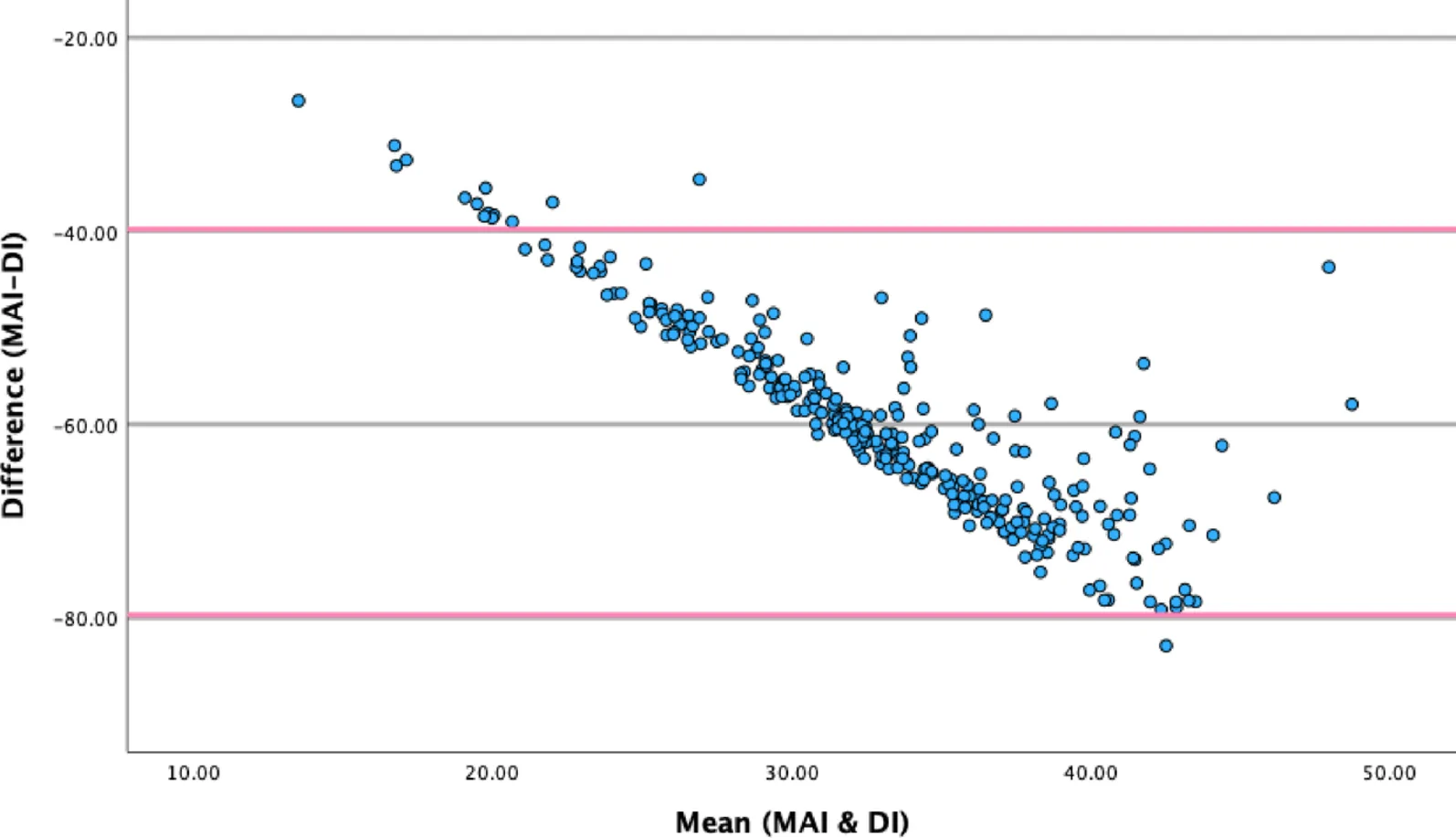
Bland-Altman plot for agreement between MAI and Food Compass (DI)

The Bland–Altman plot assessing the agreement between HEI and DI showed a relatively homogenous scatter of data points around the mean difference line (Fig. 2). However, the Bland–Altman plot assessing the agreement between the MAI and DI demonstrated a distinct pattern of proportional bias (Figs. 2 and 3).

A positive association was found between the DI and multiple subdimensions of the SHEBS, including low-fat (*r* = 0.121, *p* = 0.039), quality labels (*r* = 0.163, *p* = 0.005), healthy and balanced diet (*r* = 0.117, *p* = 0.044), and meat reduction (*r* = 0.162, *p* = 0.005). No statistically significant relationship was found between DI and seasonal food, animal welfare, or local food (*p* > 0.05) (Table 3).

**Table 3:** Correlation between DI and SHEBS factors

SHEBS Factors	*r*	*p*
Low fat	0,121	**0**,039*
Seasonal food	0,081	0,164
Animal welfare	0,110	0,060
Quality labels	0,163	**0**,005*
Local foods	0,015	0,797
Healthy and balanced diet	0,117	**0**,044*
Meat reduction	0,162	**0**,005*

Bold values indicate statistically significant results *p < 0.05

As presented in Table 4, Model 1 included age as the sole independent variable, explaining 5.4% of the variance in the total SHEBS score (R²=0.054, *p* < 0.001). Age was found to be a statistically significant predictor of SHEBS scores (β = 0.233, *p* < 0.001). In Model 2, the DI was added, resulting in a modest increase in explanatory power (R²=0.069, *p* = 0.034). DI also showed a statistically significant association with SHEBS score (β = 0.121, *p* = 0.034). Although gender, BMI, HEI, and MAI were initially included in the regression model, they were excluded from the final analysis due to a lack of statistical significance (*p* > 0.05).

**Table 4:** Regression coefficients for predictors of SHEBS score

Model		Unstandardized Coefficients		Standardized Coefficients	t	Sig.
		B	SE	Beta		
1	(Constant)	3.243	0.132		24.570	< 0.001
	Age (y)	0.017	0.004	0.233	4.090	< 0.001
2	(Constant)	2.647	0.309		8.579	< 0.001
	Age (y)	0.017	0.004	0.226	3.996	< 0.001
	DI	0.010	0.005	0.121	2.134	0.034

a. Dependent Variable: SHEBTotal

## Discussion

This study assessed the correlation between the Food Compass-derived DI and two recognized dietary indices, the HEI and MAI, while also investigating their relationship with sustainable and healthy eating behaviors.

Diet quality indices are generally based on three pillars: nutritional guidelines, nutrient intake recommendations, and dietary diversity recommendations [19]. However, NPSs were developed to evaluate the healthfulness of foods more directly with widely used examples including the “British Food Standards Agency” (FSA) in the UK, “Health Star Ratings” (HSR) in Australia and New Zealand, and “Nutri-Score” in France, Germany, Belgium, the Netherlands, and Spain Most NPSs focus on “standard” nutrients or food groups that exhibit either negative impacts (e.g., energy, saturated fat, sodium, and sugar) or beneficial impacts (e.g., fiber, fruits, and vegetables) [20]. While these systems typically focus on a limited set of nutrients or food groups with positive or negative health impacts (e.g., saturated fat, sodium, sugar, fiber, fruits, vegetables), Food Compass encompasses a broader spectrum of factors, including bioactive compounds, food additives, and the extent of food processing, offering a more multidimensional assessment of diet quality [8].

The analysis revealed moderate correlations between the novel DI, calculated using the FC NPS, and both HEI and MAI, with a stronger association observed with the latter. This suggests that these indices capture overlapping but distinct aspects of diet quality. The greater alignment with MAI relative to HEI can be attributed to the Mediterranean diet’s focus on plant-based foods, healthy fats, and minimally processed ingredients [21], which are also fundamental to the FC scoring system [8]. In contrast, the HEI was specifically designed to reflect adherence to American dietary guidelines [5], potentially overlooking the multidimensional attributes represented by FC. Bland–Altman analyses confirmed that, while there is agreement between these indices, significant differences persist, indicating that FC may offer unique insights not captured by traditional indices.

The Mediterranean diet has been thoroughly studied for its health benefits [10, 21] and may differ from scores obtained through conventional NPS. To date and based on our knowledge, there is a limited number of studies that have specifically investigated the relationship between diet quality, as assessed by an NPS, and adherence to the Mediterranean dietary pattern [9, 22]. This highlights the significance of investigating the association between the novel NPS, the FCS, and the Mediterranean diet, especially in populations where this dietary pattern is common.

This study identified a positive correlation between the DI and SHEBS, while no significant relationship was found for the HEI or MAI. Our regression analysis also indicated that age is a significant independent predictor of SHEBS, implying that older individuals may be more predisposed to adopt sustainable dietary practices, possibly due to enhanced nutritional awareness or entrenched eating habits [23]. This finding highlights the importance of considering demographic factors when evaluating the performance of dietary indices.

Moreover, beyond demographic influences, the results indicated that FC exhibits heightened sensitivity to dietary practices that adhere to sustainability principles, including a preference for plant-based, minimally processed foods and a reduction in meat consumption, which are explicitly integrated into its scoring algorithm. Plant-based diets are generally associated with reduced human health risks and improved environmental outcomes compared to animal-based diets [24].

Beyond the well-documented adverse health effects of highly or ultra-processed foods [25–27], growing evidence indicates that UPFs also have substantial environmental consequences. There are also negative environmental impacts [28, 29]. Large-scale manufacturing and intensive processing can disenfranchise small-scale farmers, undermine national food cultures, endanger animal welfare, and reduce biodiversity. Moreover, high-yield industrial production necessitates large energy inputs, which greatly increase greenhouse gas emissions and deplete resources [30, 31]. Food Compass integrates metrics on food processing and ingredients, including additives, encompassing sustainability-related aspects of diet quality that traditional indices exclude. Conversely, conventional indices like HEI and MAI predominantly emphasize nutrient adequacy and compliance with particular dietary patterns, failing to evaluate sustainability dimensions.

This underscores a unique benefit of Food Compass in conforming to contemporary dietary guidelines that advocate for both human health and environmental sustainability. Thus, a recent modeling study indicates that adopting plant-based composite diets can enhance both environmental outcomes and nutritional quality. A dietary scenario involving increased legume consumption and decreased intake of red and processed meats was projected to reduce greenhouse gas emissions by over 50%, while attaining a high Food Compass score of 74.13, underscoring its potential for facilitating sustainable dietary transitions [12]. Another study comparing the FC Score with environmental footprint data indicated that many food categories, such as fruits, legumes, and vegetables, demonstrated a strong alignment between healthfulness and environmental sustainability, whereas other categories, including grains and seafood, showed weaker correlations. This underscores the necessity of integrating nutrient profiling systems with environmental sustainability indices for a thorough assessment of diet quality [13].

However, this study has several limitations that should be acknowledged. First, the cross-sectional design makes causal links between the dietary indices and sustainable and healthy eating behaviors. Second, self-reported dietary intake may have been subject to under- or over-reporting as a result of recollection bias. Subsequent research must prioritize the validation of the Food Compass within larger and more diverse populations, ideally employing longitudinal and prospective study designs. Examining its relationship with clinical outcomes, particularly the prevalence of NCDs, would strengthen the evidence for its predictive validity and support its utility as a dietary assessment tool.

As a result, our findings suggest that Food Compass may serve as a novel, multidimensional dietary index, encompassing both traditional diet quality and sustainable eating behaviors. Food Compass incorporates elements related to both human and planetary health, offering a framework that could inform public health strategies and dietary guidelines aimed at improving long-term health and sustainability.

## Supplementary Information

Supplementary material 1

## Data Availability

The datasets used and/or analysed during the current study are available from the corresponding author on reasonable request.
