# Supplementary material for: Compatibility of the food compass with dietary indices and sustainable eating behaviors: toward a multidimensional assessment tool
Source: J Health Popul Nutr. 2026 Jul 28;45:234. doi: 10.1186/s41043-026-01410-x (PMC13693043; doi:10.1186/s41043-026-01410-x)
Supplement: Supplementary file 1 [file 41043_2026_1410_MOESM1_ESM.docx]

**Number:**

**Interviewer Name:**

**Date:**

**ASSESSMENT OF THE RELATIONSHIP BETWEEN THE FOOD COMPASS SCORE AND SUSTAINABLE AND HEALTHY EATING BEHAVIORS IN ADULTS**

Dear participant, this research is conducted to evaluate diet quality in adults using the Food Compass score and to examine its relationship with sustainable and healthy eating behaviors. All data obtained from this study will be used solely for scientific purposes, and your personal information will be kept completely confidential. Thank you for your participation.

**A. Socio-Demographic Information**

| **1. Date of birth (day/month/year)** | **…………………** | | |
| --- | --- | --- | --- |
| **2.Sex** | 1. Male | 2. Female | |
| **3.Educational Status** | | | |
| 1. Illiterate | 2.Literate | 3.Primary school | 4.Secondary school |
| 5.High school | 6.Bachelors degree | 7.Postgraduate |  |
| **4.** **Employment status** | | | |
| 1. Housewife | 2. Civil servant | 3. Paid employee | 4. Self-employed |
| 5. Worker | 6. Retired | 7. Student | 8. Other (……..) |
| **5. Marital status** | | | |
| 1. Married | | 2. Single | |
| **6. Smoking** | 1. Yes | 2. No |  |
| **7. If your answer is “YES”, for how long have you been smoking and how many cigarettes do you smoke per day?** | …………………………years……………………cigarettes/day | | |
| **8.Alcohol** | 1. Yes | 2.No | |
| **9.** **If your answer is “YES”, for how long, how often, in what amount, and what type of alcohol do you consume?** | …………years…………………day/week/month/year…………………serving/mL………………Type | | |

**B. Health Information**

| 1. **Do you have any chronic disease(s) diagnosed by a physician? If yes, please specify the disease(s).** | | 1. Yes | 2. No |
| --- | --- | --- | --- |
| Cardiovascular diseases | | 1. Yes | 2. No |
| Diabetes mellitus | | 1. Yes | 2. No |
| Hypertension | | 1. Yes | 2. No |
| Digestive system diseases (liver, gallbladder, stomach, etc.) | | 1. Yes | 2. No |
| Respiratory system diseases (lungs, etc.) | | 1. Yes | 2. No |
| Mental health problems (depression, binge eating, vomiting, night eating, etc.) | | 1. Yes | 2. No |
| Musculoskeletal system problems (osteoporosis, joint pain) | | 1. Yes | 2. No |
| Endocrine (hormonal) diseases | | 1. Yes | 2. No |
| Vitamin and mineral deficiencies (iron, vitamin B12, vitamin D deficiencies, etc.) | | 1. Yes | 2. No |
| Neurological diseases (epilepsy, migraine) | | 1. Yes | 2. No |
| Other | | …………………….. | |
| 2. **Do you use vitamin/mineral supplements?** | | 1. Yes | 2. No |
| 3. **If your answer is “Yes,” please specify the product(s) you use:** | | Reason for use ………... | |
|  |  | Duration of use ………… | |
|  |  | Frequency of use …..units/day/week/month | |
| 4. **How many meals do you usually eat per day?** | ………Main meals | …… snacks | |
| 5. **Do you skip main meals? (If no, proceed to Question 8.)** | 1.Yes | 2.No | |
| 6. **Which main meal do you usually skip?** | 1.Breakfast | 2.Lunch | 3.Dinner |
| 7. **What is your reason for skipping meals?** | 1. Unable to wake up | 2. Lack of appetite | 3.Habit |
|  | 4.Lack of tşme | 5.To lose weight | 6.Other (………..) |
| 8. **How often do you eat meals outside the home? (Foods consumed in school/workplace cafeterias or dormitories are not included.)** | 1. Every day | 2. 3-4 days per week | 3. 1-2 days per week |
|  | 4. 2 days per month | 5. 1 day per month | 6. Never |
| **9. Where do you usually prefer to eat when dining outside the home? (Please select only one option.)** | 1. À la carte / set-menu restaurant | 2. Fast food cafe | 3. Home-style restaurants |
|  | 4. School canteen | 5.Food vending machines | 6.Simit cafe |
| **10. What do you usually prefer to eat when dining outside the home? (You may select more than one option.)** | 1. Hamburger | 2. Pizza | 3. Döner |
|  | 4. Kebab / Pide / Lahmacun | 5. Fried chicken tavuk | 6. Kokoreç |
|  | 7. Simit / Pogaca / Borek | 8. Other(Please specify............................) | |
| **11.** **How many glasses of water do you usually drink?** | ………… glass | | |

**C. ANTHROPOMETRIC MEASUREMENTS AND BODY COMPOSITION**

| **Body weight (kg):** |  | **Basal metabolic rate (kcal):** |  |
| --- | --- | --- | --- |
| **Height (m):** |  | **Muscle mass (kg):** |  |
| **Waist circumference (cm):** |  | **Physical activity level:** |  |
| **Body fat (%):** |  | **Bone mass (kg):** |  |
| **Body water (%):** |  | **Visceral fat level:** |  |

**D. SUSTAINABLE AND HEALTHY EATING BEHAVIORS SCALE**

Dear participant, this questionnaire concerns your sustainable and healthy eating behaviors. Please read each question carefully and place an X in the box that best represents your response.

| **1**  Never | **2**  Very rarely | **3**  Rarely | 4  Sometimes | **5**  Often | **6**  Very often | **7**  Always |
| --- | --- | --- | --- | --- | --- | --- |

|  | **1**  Never | **2**  Very rarely | **3**  Rarely | 4  Sometimes | **5**  Often | **6**  Very often | **7**  Always |
| --- | --- | --- | --- | --- | --- | --- | --- |
| 1. I eat five portions of fruits and vegetables a day. |  |  |  |  |  |  |  |
| 2. I choose whole grains products. |  |  |  |  |  |  |  |
| 3. I choose food that contains no additives. |  |  |  |  |  |  |  |
| 4. I choose food that contains natural ingredients. |  |  |  |  |  |  |  |
| 5. I choose food that contains no artificial ingredients. |  |  |  |  |  |  |  |
| 6. Whenever possible, I buy organic food. |  |  |  |  |  |  |  |
| 7. Whenbuying food, I check certificates and quality marks on labels. |  |  |  |  |  |  |  |
| 8. I choose products with clear information/indication on country of origin. |  |  |  |  |  |  |  |
| 9. I don’t waste food. |  |  |  |  |  |  |  |
| 10. I reuse leftovers from food. |  |  |  |  |  |  |  |
| 11. I buy regional food. |  |  |  |  |  |  |  |
| 12. I eat seasonal fruits and vegetables. |  |  |  |  |  |  |  |
| 13. In season,I shop at farmer’s market. |  |  |  |  |  |  |  |
| 14. I avoid sugary drinks. |  |  |  |  |  |  |  |
| 15. I limit my salt usage. |  |  |  |  |  |  |  |
| 16. I keep food such as cakes, sweets and chocolate to an occasional treat. |  |  |  |  |  |  |  |
| 17. I choose food that keeps me healthy. |  |  |  |  |  |  |  |
| 18. I choose food that is nutritious. |  |  |  |  |  |  |  |
| 19. I try to have a balanced diet. |  |  |  |  |  |  |  |
| 20. I choose food that is produced in an environmental friendly way. |  |  |  |  |  |  |  |
| 21. I buy fruits and vegetables directly from the farmer. |  |  |  |  |  |  |  |
| 22. Whenever possible, I choose fruits and vegetables from my own allotments(plots). |  |  |  |  |  |  |  |
| 23. I buy locally produced foods. |  |  |  |  |  |  |  |
| 24. I try to eat as much pulses as possible in order to reduce meat consumption. |  |  |  |  |  |  |  |
| 25. Pulses replace meat in my cooking. |  |  |  |  |  |  |  |
| 26. Whenever possible I buy free range eggs. |  |  |  |  |  |  |  |
| 27. I avoid buying battery eggs. |  |  |  |  |  |  |  |
| 28. Whenever possible,I buy fish from sustainable fishing. |  |  |  |  |  |  |  |
| 29. I avoid food products containing lots of fat. |  |  |  |  |  |  |  |
| 30. I choose low fat food products. |  |  |  |  |  |  |  |
| 31. Whenever possible, I choose low fat food products. |  |  |  |  |  |  |  |
| 32. I try not to throw away food. |  |  |  |  |  |  |  |

**E. 24-HOUR DIETARY RECALL**

| **MEALS** | **FOODS** | **AMOUNT** | **INGREDIENTS** |
| --- | --- | --- | --- |
| **BREAKFAST** |  |  |  |
| **SNACK** |  |  |  |
| **LUNCH** |  |  |  |
| **SNACK** |  |  |  |
| **DINNER** |  |  |  |
| **SNACK** |  |  |  |
